# Interaction Between Prematurity and the MAOA Gene on Mental Development in Children: A Longitudinal View

**DOI:** 10.3389/fped.2020.00092

**Published:** 2020-03-09

**Authors:** Nai-Jia Yao, Wu-Shiun Hsieh, Chyi-Her Lin, Ching-Ing Tseng, Wan-Yu Lin, Po-Hsiu Kuo, Yen-Ting Yu, Wei J. Chen, Suh-Fang Jeng

**Affiliations:** ^1^School and Graduate Institute of Physical Therapy, College of Medicine, National Taiwan University, Taipei, Taiwan; ^2^Department of Pediatrics, National Taiwan University Hospital, Taipei, Taiwan; ^3^Department of Pediatrics, National Cheng Kung University Hospital, Tainan, Taiwan; ^4^Centers of Genomic and Precision Medicine, National Taiwan University, Taipei, Taiwan; ^5^Institute of Epidemiology and Preventive Medicine, College of Public Health, National Taiwan University, Taipei, Taiwan; ^6^Research Center for Genes, Environment and Human Health, National Taiwan University, Taipei, Taiwan; ^7^Department of Psychiatry, National Taiwan University Hospital and College of Medicine, National Taiwan University, Taipei, Taiwan; ^8^Physical Therapy Center, National Taiwan University, Taipei, Taiwan

**Keywords:** dopamine, genetics, child development, cognitive development, prematurity, longitudinal analysis

## Abstract

This study aimed to examine the association of dopamine-related genes with mental and motor development and the gene-environment interaction in preterm and term children. A total of 201 preterm and 111 term children were examined for their development at 6, 12, 18, 24, and 36 months and were genotyped for 15 single-nucleotide polymorphisms (SNPs) in dopamine-related genes (*DRD2, DRD3, DAT1, COMT*, and *MAOA*). An independent sample of 256 preterm children was used for replication. Since the developmental age trends of preterm children differed from those of term children, the analyses were stratified by prematurity. Among the 8 SNPs on the *MAOA* gene examined in the whole learning sample, the results of linkage disequilibrium analysis indicated that they were located in one block (all D′ > 0.9), and rs2239448 was chosen as the tag (r^2^ > 0.85). In the analysis of individual SNPs in each dopamine-related gene, the tag SNP (rs2239448) in *MAOA* remained significantly associated with the mental scores of preterm children for the interaction with age trend (*p* < 0.0001; largest effect size of 0.65 at 24 months) after Bonferroni correction for multiple testing. Similar findings for rs2239448 were replicated in the independent sample (*p* = 0.026). However, none of the SNPs were associated with the motor scores of preterm children, and none were related to the mental or motor scores of term children. The genetic variants of the *MAOA* gene exert influence on mental development throughout early childhood for preterm, but not term, children.

## Introduction

Preterm children with very low birth weight (VLBW, birth body weight <1,500 g) face an elevated risk of neurodevelopmental impairments throughout childhood (1–3). Both genetic and environmental factors may contribute to these developmental consequences (4, 5). Recent evidence has shown that the association of preterm birth with severe brain structural abnormalities, congenital disease (6, 7), and developmental problems (8) might be accounted for by a common genetic background. The dopamine system (DA) is a major neurotransmitter in the extrapyramidal system of the brain that involves motor control, endocrine function, cognition, reward system and behavior (9). Dopamine-related genes have long been implicated in child development (9, 10). Certain single nucleotide polymorphisms (SNPs) of dopamine-related genes (e.g., dopamine receptor 2 [*DRD2*] and *DRD3* for DA release, dopamine transporter [*DAT*] for DA clearance, monoamine oxidase A [*MAOA*] and catechol-O-methyltransferase [*COMT*] for DA degradation) have been associated with neurodevelopmental disorders in children. Specifically, several SNPs in *DAT1* (rs27072 and rs2550948) and *MAOA* (i.e., rs12843268, rs2072744, rs5905859, rs3027400, rs2235186, rs2235185, rs2239448, and rs3027407) have been associated with attention deficit hyperactivity disorder (ADHD) (11–16); some SNPs in *DRD2* (rs1800497) and *DRD3* (rs167771) have been related to child affective problems, obesity, working memory impairment, or autism spectrum disorders (ASD) (17–22); and some SNPs in *COMT* (rs4818, rs4680, and rs2075507) have been associated with ASD, aggressive behavior, verbal inhibition and poor working memory in school-aged children (23–25).

A challenge in research on child development is how to incorporate longitudinal data from repeated measurements (26). To date, few studies on the role of dopamine-related genes have adopted a longitudinal view that incorporates repeated measurements of development. Furthermore, existing studies have mainly been conducted among Caucasian populations on more severe developmental morbidities. Whether dopamine-related genes are involved in more common forms of developmental variation in preterm or term children, particularly among non-Caucasian populations, remains poorly understood.

To address the gap in the literature, we turned to a prospective follow-up study of preterm children and their term counterparts. The specific aims of this study were to (1) examine the association of dopamine-related genes with mental and motor development in preterm children with VLBW and term children with normal birth weight at 6, 12, 18, 24, and 36 months of age, (2) examine whether environmental factors (preterm/term birth) interact with dopamine-related genes in children's mental and motor development, and (3) replicate the findings in an independent sample of children.

## Materials and Methods

### Participants

The participants of the learning sample consisted of 201 preterm children with VLBW and 111 term children who were born in or admitted to three hospitals in northern Taiwan during the time periods of 1995–1997 (Cohort I), 2002–2004 (Cohort II), and 2006–2008 (Cohort III). Meanwhile, the replication sample had 256 preterm children with VLBW recruited during the time period of 2012–2014 (Cohort IV) from northern and southern Taiwan. More detailed descriptions of the recruitment are provided in the Supplementary Methods and Figure S1. Briefly, the inclusion criteria for VLBW preterm children from all cohorts were birth weight <1,500 g, gestational age <37 weeks, and the absence of congenital abnormalities and severe neonatal diseases. The selection criteria for term children included gestational age within 38–42 weeks, birth weight ≥ 2,500 g, and the absence of congenital abnormality and perinatal disease. All mothers were Taiwanese citizens, were over 18 years old, and had no history of psychiatric disorders or drug or alcohol abuse. All of the participants were from different families.

This study was approved by the Research Ethics Committee of National Taiwan University Hospital and the Institutional Review Board of National Cheng Kung University Hospital, and written informed consent was obtained from parents. Both the Cohort I and II studies were observational studies, while the Cohort III and IV studies were randomized controlled trials (URL: https://clinicaltrials.gov/, identifiers: NCT00173108 and NCT00946244 for Cohort III, and NCT01807533 for Cohort IV, National Taiwan University Hospital). Preterm children in Cohorts III and IV were randomly assigned to the intervention group [clinical-based intervention group and home-based intervention group in Cohort III (27); the family-centered intervention group in Cohort IV (28)] and the usual-care group (Cohorts III and IV). Children in the intervention group received 5 in-hospital and 7 after-discharge intervention sessions from hospitalization to 12 months of corrected age that emphasized environmental modulation, feeding support, massage, child developmental skills, parental support and education, and dyadic interaction activities. Children in the usual-care group received standard care. Randomizations were computer-generated and stratified by gestational age and hospital, with the sequence kept in a locked file and concealed from the parents, medical staff, and outcome examiners. However, the parents and the intervention providers were aware of group allocation. All methods were performed in accordance with the relevant guidelines and regulations.

### Measurements

Children in Cohorts I to III had their perinatal and demographic data collected via chart review and parental interviews, and their developmental outcomes were evaluated at 6, 12, 18, 24, and 36 months of age using the Bayley Scales of Infant Development−2nd Edition (BSID-II) (29). Because the BSID-II was revised into the Bayley Scales of Infant and Toddler Development−3rd Edition (Bayley-III) (30) in 2006, both versions were administered to children in Cohort III at five time points (6, 12, 18, 24, and 36 months). For children in Cohort IV, the Bayley-III was administered at four time points (6, 12, 24, and 36 months), and the BSID-II was additionally administered at two time points (24 and 36 months). Because the replication sample had no BSID-II scores at 6 and 12 months, their Bayley-III scores at these ages were transformed into BSID-II scores using a linear regression procedure (31) (details in Supplementary Methods).

### Genetic Analyses

A buccal cell sample was collected from the cheeks of each child using the Catch-All swabs of the BuccalAmp™ DNA Extraction Kit (Epicenter Biotechnologies, WI, USA). Genomic DNA was extracted from the buccal cell samples following the standard protocol of a commercial kit, the QIAamp Mini Kit (Qiagen, Chatsworth, CA, USA). A total of 15 SNP markers, including 1 in *DRD2* (rs1800497) (19), 1 in *DRD3* (rs167771) (17), 2 in *DAT1* (rs27072 and rs2550948) (12), 3 in *COMT* (rs4818, rs4680; and rs2075507) (23, 24), and 8 in *MAOA* (i.e., rs12843268, rs2072744, rs5905859, rs3027400, rs2235186, rs2235185, rs2239448, and rs3027407) (13), were selected for genotyping using TaqMan analysis and a 7900HT Fast Real-Time PCR System (Applied Biosystems, Foster City, CA, USA). Quality control of the genotyping included duplications, a negative control, and a call rate > 95%. Although there is a well-known variant number of tandem repeats on *MAOA* (32), we did not include it in this study, since its genotyping requires different designs and sophisticated quality control.

None of the genotypes for any of the 15 markers showed significant deviation from Hardy-Weinberg equilibrium, as calculated using PLINK (33) (version 1.07, http://pngu.mgh.harvard.edu/purcell/plink/), with *p* > 0.001 defined as significant departure in either preterm or term children. The *MAOA* gene was examined in females only because males are hemizygous at X-linked loci.

### Statistical Analysis

Both the Cohort III and IV were superiority randomized controlled trials. Based on the cognitive and motor development for preterm children for a statistical power of 80%, with an attrition rate of 20% and an alpha level of 0.05, the estimated sample size in the Cohort III study was 43 in each group of preterm children (34). For the Cohort IV study, for a power of 80% with a 20% attrition and an alpha level of 0.05, the estimated sample size in each group was 78 for the 24-month cognitive outcome and 115 for the 24-month motor outcome (27).

We used the QUANTO software (version 1.2.4, http://hydra.usc.edu/gxe) to estimate the overall sample size for the investigation of gene-environment interaction effects in both learning sample and replication sample. To achieve a power of 80%, with continuous outcome and independent individuals design, and an effect size as reported by Babineau et al. (35) for the interaction effect of *5-HTTLPR* gene and environmental factors on child behavior scores (R^2^ = 0.04 to 0.06, from 3 to 36 months of age) as well as an attrition rate of 20%, an estimated sample size of 153 to 231 children were required.

The power analysis of our existing sample size of 111 term children and 201 preterm children with an alpha level at 0.05 using G^*^Power software (version 3.1.9.4, Germany) indicated a power of 60–100% for an effect size ranging from 0.11 to 0.65, derived from the standardized mean differences in mental score between the two genotype groups of rs2239448 at 6, 12, 18, 24, and 36 months of age.

Group comparisons were conducted using analysis of variance for continuous variables and chi-square tests for categorical variables. The alpha level was set at 0.05. Because some homozygous genotypes had a small number of individuals for certain SNPs, we merged two adjacent genotypes to adopt a dominant or recessive model as indicated. The pattern of linkage disequilibrium (LD) was examined using Haploview (36).

The relations of the genotypes of individual SNPs to mental and motor raw scores were first examined in the learning sample. We started with a PROC GLIMMIX model with main effects of preterm birth, genotype, and age trend (measured at 5 time points) and their pairwise two-way interactions and three-way interaction with a random intercept and adjustment for covariates, including cohort sources, intervention (yes or no), and sex. Analysis of preterm children's data was additionally adjusted for the effect of gestational age. The cohort differences in developmental scores are described in more detail in Table S1. An unstructured covariance structure was selected for the repeated observations based on the Akaike information criterion and the Bayesian information criterion (37). The significance level of the *p*-value obtained from the learning sample was adjusted according to the Bonferroni correction (38, 39). In correcting for multiple testing, we considered the *p*-values of the main effect variables and the interactions together. If the three-way interaction was not significant, the model was refitted by deleting the interaction term from the model. Then, the correction for multiple testing was conducted again for this refitted model without the three-way interaction. Under this circumstance, we conducted stratified analyses to examine the effect of age trend, genotype, and their interaction separately for preterm and term children, with correction for multiple testing within each stratum.

To examine the cumulative effect of significant genetic markers on developmental scores, we used the regression coefficients from mixed-effects models as weights to generate a summarized genetic risk score. The scores were compared using mixed-effects models of longitudinal data at five time points to examine whether the cumulative effects existed or not. Similar correction for multiple testing was conducted for this part of the analysis using the Bonferroni correction (38, 39) by considering the *p*-values of the main effect variables and the interactions together. All statistical analyses were conducted using Statistical Analysis Software (SAS, version 9.3, SAS Institute, Cary, NC, USA).

## Results

The birth and demographic characteristics of the participating children are presented in Table 1. In the learning sample, preterm children had lower gestational age, birth body weight, parental education, and developmental scores and were more likely to be in Cohort III than were term children (all *p* < 0.05). The results of LD analysis for the 8 SNPs of the *MAOA* in the whole learning sample indicated that they were located in one block (all *D*′> 0.9) (Figure S2), and rs2239448 was therefore chosen as the tag (*r*^2^ > 0.85).

**Table 1:** Birth and demographic characteristics of preterm and term children.

**Characteristics**	**Learning sample (Cohort I-III)**		**Replication sample (Cohort IV)**
	**Preterm (*N* = 201)**	**Term (*N* = 111)**	**Preterm (*N* = 256)**
	**Number (%)**	**Number (%)**	**Number (%)**
Male sex	99 (49%)	59 (53%)	129 (50%)
Maternal education^a^			
College or above	126 (63%)	83 (82%)	197 (77%)
High school below	75 (37%)	18 (18%)	59 (23%)
Paternal education^a^			
College or above	120 (60%)	83 (81%)	192 (75%)
High school or below	81 (40%)	19 (19%)	64 (25%)
Cohort*			
I	27 (14%)	38 (34%)	–
II	39 (19%)	24 (22%)	–
III	135 (67%)	49 (44%)	–
Intervention type			
Intervention	95 (47%)	–	125 (49%)
Standard care	106 (53%)	–	131 (51%)
	**Mean (SD)**	**Mean (SD)**	**Mean (SD)**
Gestational age (weeks)^a^	29.5 (2.9)	39.1 (1.0)	29.6 (2.6)
Birth body weight (g)^a^	1,115.5 (265.0)	3,287.7 (380.7)	1,112.1 (260.1)
Bayley mental raw scores^a^			
6 months	57.6 (4.4)	59.1 (2.7)	57.5 (2.9)
12 months	82.4 (4.1)	85.1 (3.5)	83.1 (2.5)
18 months	104.8 (6.2)	108.5 (5.5)	–
24 months	127.3 (8.0)	131.2 (7.0)	129.3 (7.3)
36 months	150.7 (6.4)	157.3 (5.2)	149.8 (7.7)
Bayley motor raw scores^a^			
6 months	35.0 (4.1)	36.4 (3.5)	–
12 months	59.1 (2.9)	61.5 (3.1)	–
18 months	71.4 (3.1)	72.8 (2.4)	–
24 months	81.0 (3.6)	83.0 (3.2)	–
36 months	97.0 (3.9)	100.8 (3.5)	–

a *p < 0.05 in comparing preterm children with term children in the learning sample using t-tests or analysis of variance for continuous variables and chi-square tests for categorical variables*.

The distributions of the 8 SNPs (rs2239448 as the tag of *MAOA*) genotyped in this study are displayed in Table S2. We adopted a dominant model for the designated allele 1, which was determined by collapsing two adjacent genotypes if their distributions were closer than the remaining genotypes or one homozygous group having a very small number (one in *DRD3* and two in *DAT1*).

### Effects of Genetic Factors and Prematurity on Longitudinal Child Development

The results of the mixed-effects model analysis using a full model for the mental score with Bonferroni correction for 56 *p-*values are displayed in Table S3. Because the three-way interaction was not significant in each of the 8 SNPs, we then removed the three-way interaction and refitted the model (Table S4). Regarding the main effect, age trend and preterm birth were highly significant even after Bonferroni correction for 48 *p-*values, and genotype was not. In terms of interaction, the preterm × age trend was significant for all SNPs, but both the genotype × age trend and genotype × preterm interactions were significant only for the *MAOA* rs2239448, though the latter was not significant after Bonferroni correction. One possible reason for not reaching significance for the genotype × preterm interaction is the relatively small sample size for the term group (*N* = 111) vs. the preterm group (*N* = 201).

Because there was no three-way interaction and the sample was recruited separately for preterm and term children, we then conducted stratified analyses by preterm birth to examine the effect of age trend, genotype, and their interaction. For the stratum of preterm children's mental score (Table 2), the effect of age trend was significant for all SNPs, but the effect of genotype and genotype × age trend were significant only for the *MAOA* rs2239448 after Bonferroni correction for 24 *p-*values and adjustment for the effect of gestational age, cohort sources, intervention and sex. For illustration, the mean differences between two genotype groups at the five time points are also displayed for each SNP in Table 2 (more detailed mental score distributions are presented in Table S5). Of note, *MAOA* rs2239448 showed the smallest genotype effect (*p* = 0.0032) and genotype × age trend interaction effect (*p* < 0.0001), yet only the latter remained statistically significant after Bonferroni correction. The effect size for *MAOA* rs2239448 (i.e., mean differences between the two genotype groups divided by the standard deviation of preterm children) in relation to the mental score was 0.11, 0.27, 0.51, 0.65, and 0.39 at 6, 12, 18, 24, and 36 months of age, respectively.

**Table 2:** Relations of genotypes with the mental raw scores at 6, 12, 18, 24, and 36 months of age in preterm children (cohort, intervention, sex, and gestational age are treated as covariates).

**Gene**	**SNP (allele 1/2)**	**Difference in mental score (presence of allele 1 - absence of allele 1)**					**Age trend**		**Genotype**		**Genotype × Age trend**	
		**6 months**	**12 months**	**18 months**	**24 months**	**36 months**	***F*-value**	***p***	***F*-value**	***p***	***F*-value**	***p***
**Learning sample**												
DRD2	rs1800497 (G/A)	1.78	0.56	−0.92	0.18	1.56	6,798	<0.0001^b^	1.39	0.24	1.34	0.25
DRD3	rs167771 (G/A)^a^	−0.20	0.91	1.78	1.95	1.46	11,707	<0.0001^b^	1.74	0.19	1.47	0.21
DAT1	rs27072 (T/C)^a^	−1.60	0.16	−0.58	−0.05	−0.35	12,396	<0.0001^b^	2.84	0.09	0.18	0.94
DAT1	rs2550948 (T/C)^a^	−0.01	0.10	0.33	0.92	0.88	10,966	<0.0001^b^	1.09	0.29	0.56	0.69
COMT	rs4818 (C/G)	−0.29	0.03	0.09	1.33	0.82	9,005	<0.0001^b^	0.00	0.99	0.92	0.45
COMT	rs4680 (G/A)	−1.02	0.09	−1.67	−1.75	−0.91	8,548	<0.0001^b^	2.85	0.09	0.88	0.47
COMT	rs2075507 (T/C)	−0.50	0.50	−0.95	−1.50	1.45	5,389	<0.0001^b^	0.22	0.64	1.07	0.37
MAOA	rs2239448 (T/C)	0.50	1.09	3.16	5.23	2.49	11,026	<0.0001^b^	8.76	0.0032	8.27	<0.0001^b^
**Replication sample**												
MAOA	rs2239448 (T/C)	−0.04	0.84	–	3.11	1.38	8,140	<0.0001	4.19	0.041	3.12	0.026

*The BSID-II scores at 6 and 12 months of age were transformed from Bayley-III, and the significance level was set as 0.05 in the replication sample. DRD2/3, dopamine D2/D3 receptors; DAT1, dopamine transporter 1; COMT, catechol-O-methyltransferase; MAOA, monoamine oxidase A*.

a *Determined by collapsing two adjacent genotypes due to one homozygous group having a very small number*.

b *The significance levels reached the threshold of Bonferroni correction for 24 p-values in the learning sample*.

For the stratum of term children's mental scores (Table S6), the effect of age trend was significant for all SNPs, but the SNPs showed neither genotype nor genotype × age trend effects after Bonferroni correction for 24 *p-*values.

For the motor scores, we first used a full model containing the three main variables, all of their possible interactions, and covariates (Table S7). Regarding the main effect, age trend and preterm birth were significant, while genotype was not. In terms of interactions, the preterm × age trend was significant, which was also expected according to a previous study (26), but the genotype × age trend and genotype × preterm interactions and the three-way interaction were not significant. Even if we refitted the model without the three-way or two-way interactions, the results still indicated that only preterm, age trend, and preterm × age trend were significant.

### Replicating the Finding in *MAOA*

To examine the robustness of the influence of *MAOA* rs2239448 and its interaction with age trend on the mental scores of preterm children, we further genotyped this SNP in an independent sample of 256 preterm children. *MAOA* rs2239448 had a significant main effect (*p* = 0.041) as well as a significant interaction effect with age trend (*p* = 0.026) (Table 2, bottom row). For *MAOA* rs2239448 in this replication sample, its effect size with the mental score was −0.01, 0.34, 0.43, and 0.18 at 6, 12, 24, and 36 months of age, respectively. Of note, the SD at 12 months of age (2.5) was derived from the Bayley-III, whereas the developmental data at 36 months of age (7.7) was derived from the BSID-II, resulting in a larger effect size for the former even though it had a smaller group difference than the latter.

**Figure 1:**
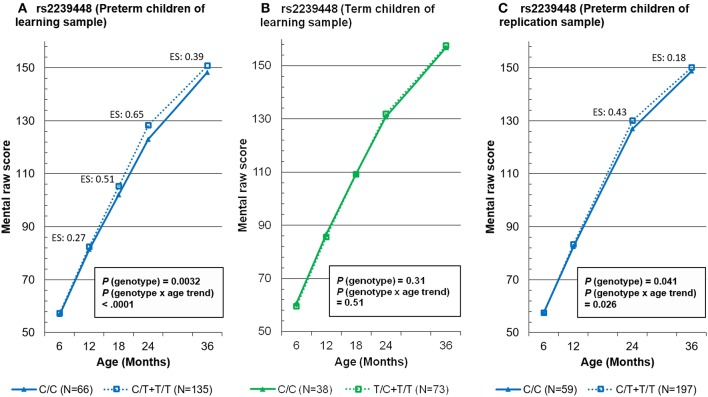
Mixed-effects model-based mental raw score at 5 time points by the genotypes of *MAOA* tag rs2239448 in preterm and term children in the learning sample (**A,B**, respectively) and in preterm children in the replication sample **(C)**. ES, effect size.

To summarize the findings for *MAOA* rs2239448, the mean mental scores at five time points for the two genotype groups (presence of allele 1 vs. absence of allele 1) are depicted separately for the preterm children in the learning sample (Figure 1A), the term children in the learning sample (Figure 1B), and the preterm children in the replication sample (Figure 1C).

Then, the most significant *MAOA* rs2239448 was combined sequentially with seven other markers according to the ascending order of *p*-value, and the cumulative genetic dose was subjected to mixed-effects model analysis after Bonferroni correction for 56 *p-*values (Table S8). Nevertheless, the combination of rs2239448 with other SNPs did not lead to any increase in the effect estimates regarding genotype, i.e., the main effect of genotype, genotype × age trend, genotype × preterm, and genotype × preterm × age trend.

## Discussion

In this prospective study, we evaluated the potential influence of 15 dopamine-related genetic variants on developmental scores using mixed-effects models of longitudinal data to examine three main-effects variables (preterm, genotype, and age trend) and their possible interactions. After removing the three-way interaction due to its lack of statistical significance, the three two-way interactions were significant for the *MAOA* variant. After stratification by preterm birth, the effect of the genotype × age trend remained significant only for preterm children's mental scores for the variant of *MAOA* rs2239448 after Bonferroni correction. Furthermore, the influence of *MAOA* rs2239448 (genotype main effect and genotype × age trend interaction) in preterm children was successfully replicated in an independent sample. Nevertheless, a combination of *MAOA* rs2239448 with the SNPs of other dopamine-related genes failed to improve the association. These findings provide insightful information on the gene (*MAOA* rs2239448) × environment (preterm birth) interaction regarding the age trend of mental development.

As expected, preterm children demonstrated persistently lower mental and motor scores than their term peers did from 6 to 36 months of age. Such results were in line with previous findings indicating that preterm children showed poorer motor and mental development than their term counterparts did in their follow-up to 24 months of age (27).

Among the variants of dopamine-related genes examined, only those of *MAOA* exhibited an association with the mental scores of preterm children at different ages, with a small to medium effect size in the learning sample and a small effect size in the replication sample. For example, the preterm children with two genotypes on the *MAOA* rs2239448 had the largest difference in mental scores of 5.23 (i.e., a medium effect size of 0.65) in the learning sample and of 3.11 (i.e., a small effect size of 0.43) in the replication sample at 24 months of age. Although these *MAOA* variants have been found to be associated with some childhood-onset mental disorders, including ADHD (13) and ASD (40), this study is the first to demonstrate that the *MAOA* variants exert influence on the mental development of preterm children, providing further support for the role of *MAOA* implicated in prior animal studies (41).

On the other hand, our findings showed no association of dopamine-related genes with mental development in term children. One possibility is that the dopamine-related brain functions of term children have less variation than those of preterm children (42, 43). Hence, many previous studies have consistently reported no association between variants of dopamine-related genes and cognition in healthy children and adolescents (44, 45). In contrast, some prior studies found that the influence of dopamine-related genes could be detected only under certain environmental contexts, for example, the influence of *MAOA* on antisocial problems moderated by childhood maltreatment (46, 47), the influence of *5-HTT* on dysregulation in children moderated by prenatal depression in mothers (35), and the influence of *5-HTT* behavioral development in children moderated by child care quality (48). Therefore, the effect of dopamine-related genes on mental development may be very small unless the children underwent an adverse developmental environment, e.g., preterm birth in this study. Thus, our findings implied that preterm children are more vulnerable to the influence of *MAOA* on mental development.

A combination of the *MAOA* rs2239448 with the SNPs of other dopamine-related genes in this study did not lead to a stronger association with mental development in preterm children, as indicated in a previous study (49). One possibility is that our chosen genetic variants of other dopamine-related genes were mainly based on Caucasian populations and not on the most informative alleles in our study population.

Our failure to find any association of the variants of dopamine-related genes with motor development in preterm and term children is, to some extent, not surprising, given that our target markers were chosen based on studies focusing on childhood-onset diseases such as ADHD and ASDs. Future studies on child motor development need to consider genetic markers that may have associations with other motor-related diseases or functions.

Our findings indicate that prematurity in children with the absence of the T allele of *MAOA* rs2239448 is associated with slower mental development. In practice, the *MAOA* rs2239448 may be used to identify preterm children who are vulnerable and can benefit from early intervention to help them overcome the slowing pace of mental development. It is also warranted to investigate whether the *MAOA* variants moderate the effect of intervention in preterm children and help develop personalized interventions for preterm children.

This study has the following strengths: (1) the incorporation of a longitudinal design with follow-ups at 6, 12, 18, 24, and 36 months on both the mental and motor development of preterm and term children and (2) the successful replication of the robust results in an independent sample of preterm children. However, several limitations are important to note. First, because preterm children with severe brain damage or neonatal diseases were excluded from this study, our findings may not be generalizable to those with more severe developmental disorders. Second, this study consisted of participants from several cohorts established in different years. While clinical practice and environmental exposure might vary across time, we treated cohort membership as a covariate to control for potential confounding effects of birth year. Some information was not collected for some cohorts, such as socioeconomic status for Cohort I and Cohort II. The failure to include socioeconomic status as a covariate in our analysis might render our adjustment for potential confounders inadequate. Third, our study focused only on certain candidate SNPs selected from previous genome-wide association studies. Future research may explore the *MAOA* variable number of tandem repeats for more genetic information. Finally, the BSID-II scores at both 6 and 12 months of age in the replication sample of Cohort IV were transformed from the Bayley-III scores via regression modeling. This might not overcome the difference between the two, e.g., a smaller standard deviation of the Bayley-III scores at the age of 12 months than its counterparts of BSID-II scores.

In conclusion, this prospective study of preterm and term children demonstrated that the *MAOA* rs2239448 variants were significantly associated with the mental scores of preterm children for the main effect and its interaction with the age trend. Similar findings for *MAOA* rs2239448 were replicated in an independent sample of preterm children. However, none of the SNPs were associated with the motor scores of preterm children, and none were related to the mental or motor scores of term children. Our results have shed new light on the genetic influences of *MAOA* on mental development in preterm children and have implications for intervention.

## Data Availability Statement

The raw data supporting the conclusions of this article will be made available by the authors, without undue reservation, to any qualified researcher.

## Ethics Statement

The studies involving human participants were reviewed and approved by the Ethics Committee of National Taiwan University Hospital (Identifier on ClinicalTrials.gov: NCT00173108, NCT00946244, and NCT01807533). Written informed consent to participate in this study was provided by the participants' legal guardian/next of kin.

## Author Contributions

N-JY conceptualized and designed the medical data collection and analysis procedures, contributed to child developmental data collection, carried out the genetic data collection and genotyping experiment, and drafted the initial manuscript. W-SH and C-HL supervised case enrollment and medical data collection in two different hospitals. C-IT carried out the DNA extraction and assisted genotyping experiment. W-YL and P-HK contributed to the statistical analysis and interpretation of the data. Y-TY helped with child developmental data collection. WC supervised the genotyping experiment and statistical analysis, contributed to the interpretation of data, and revised the manuscript critically. S-FJ designed the study, procured the grant support, coordinated all data collection of all the hospitals, supervised the child developmental data collection, and critically revised the manuscript. All authors reviewed and approved the manuscript.

### Conflict of Interest

The authors declare that the research was conducted in the absence of any commercial or financial relationships that could be construed as a potential conflict of interest.
