# Supplementary material for: Interaction Between Prematurity and the MAOA Gene on Mental Development in Children: A Longitudinal View
Source: Front Pediatr. 2020 Mar 9;8:92. doi: 10.3389/fped.2020.00092 (PMC7075243; doi:10.3389/fped.2020.00092)
Supplement: Supplementary file 1 [file Data_Sheet_1.docx]

**Supplementary Material**

**Title:**

Interaction of Prematurity with MAOA Gene on Mental Development in Children: A Longitudinal View

**Authors:**

Nai-Jia Yao^1^, Wu-Shiun Hsieh^2^, Chyi-Her Lin^3^, Ching-Ing Tseng^4^, Wan-Yu Lin^5^, Po-Hsiu Kuo^5,6^, Yen-Ting Yu^1^, Wei J. Chen^4,5,7*^, Suh-Fang Jeng^1,8*^

*** Correspondence:**

Wei J. Chen and Suh-Fang Jeng

wjchen@ntu.edu.tw; jeng@ntu.edu.tw

**Supplementary Methods**.

**Supplementary tables and figures**

**Table S1**. Comparison of developmental scores of cohorts in preterm and term children, respectively

**Table S2**. Distribution for the 15 SNPs of dopamine-related genes

**Table S3**. Relations of genotype, preterm birth and age trend with the mental raw scores in the learning sample using three-way models (cohort, intervention and sex treated as covariates)

**Table S4**. Relations of genotype, preterm birth and age trend with the mental raw scores in the learning sample using two-way models (cohort, intervention and sex treated as covariates)

**Table S5**. Mental raw scores in preterm and term children in learning sample at 6, 12, 18, 24 and 36 months of age

**Table S6**. Relations of genotypes with the mental raw scores at 6, 12, 18, 24 and 36 months of age in term children of the learning sample (cohort, intervention and sex are treated as covariates)

**Table S7**. Relations of genotype, preterm birth and age trend with the motor raw scores in the learning sample using three-way models (cohort, intervention and sex treated as covariates)

**Table S8**. Exploring the combinations of rs2239448 with other SNPs on the analysis of genotype, preterm birth, and age trend with the mental raw scores in the learning sample using three-way models (cohort, intervention, sex and gestational age treated as covariates)

**Figure S1** Flow chart of allocation and follow-up in preterm children and term children among each cohort

**Figure S2** Haplotype and linkage disequilibrium plot of SNPs in *MAOA* gene in Cohort I-III (N = 312)

**Supplementary Methods**.

**Participants**

The participants of the learning sample were preterm children with VLBW and term children who were born or admitted at three hospitals in northern Taiwan, including National Taiwan University Hospital, Branch for Women and Children at Taipei City Hospital, and MacKay Memorial Hospital, during the time periods of 1995-1997 (Cohort I), 2002-2004 (Cohort II), and 2006-2008 (Cohort III), respectively. Meanwhile, the replication sample was based on a new cohort of preterm children with VLBW recruited during the time period of 2012- 2014 (Cohort IV) from northern and southern Taiwan including National Taiwan University Hospital, MacKay Memorial Hospital, and National Cheng Kung University Hospital. The inclusion criteria for VLBW preterm children from all cohorts were birth weight < 1,500 g, gestational age < 37 weeks, and absence of congenital abnormality and severe neonatal diseases. The selection criteria for term children included gestational age within 38-42 weeks, birth weight ≥ 2,500 g, and absence of congenital abnormality and perinatal disease. All mothers were Taiwanese citizens, aged over 18 years, and had no history of psychiatric disorders or drug or alcohol abuse. All of the participants were from different families.

The learning sample consisted of 201 preterm children (27 from Cohort I, 39 from Cohort II, and 135 from Cohort III) and 111 term children (38 from Cohort I, 24 from Cohort II, and 49 from Cohort III); whereas, the replication sample contained 256 preterm children in Cohort IV (more detail in Figure S1). Preterm children in Cohorts I and II received standard developmental care that included child-focused intervention during hospitalization and neonatal clinic visits for health care; preterm children in Cohort III and Cohort IV were randomly assigned into the control group for standard developmental care and the intervention groups for child-, parent-, and dyad-focused services lasting from birth until 12 months of age. (1, 2)

**Measurements**

Children in Cohort I to III had their perinatal and demographic data collected via chart review and parental interview, and their developmental outcomes evaluated at 6, 12, 18, 24, and 36 months of age using the Bayley Scales of Infant Development – 2^nd^ Edition (BSID-II) (3). Because the BSID-II was revised into the Bayley Scales of Infant and Toddler Development – 3^rd^ Edition (Bayley-III) (4) in 2006, children in Cohort III were administered with both versions at five time points (6, 12, 18, 24, and 36 months). For children in Cohort IV, the Bayley-III was administered at four time points (6, 12, 24, and 36 months) and the BSID-II was additionally administered at two time points (24 and 36 months). Some developmental assessments were missing due to parental refusal, incompatible schedule, moving, or dropping out. All the examiners were blind to the children’ group status and achieved high reliability against the standard of an experienced examiner (correlation coefficients 0.82-0.99) before conduction of assessment in the study.

It is noteworthy that the BSID-II and Bayley-III have different scoring items. The BSID-II includes 178 items for mental and 111 items for motor scales (3), whereas the Bayley-III contains 91 items for cognitive, 97 items for language, and 138 items for motor scales (4). Because the replication sample had no BSID-II scores at 6 and 12 months, their Bayley-III scores at these ages were transformed into BSID-II scores according to the following procedure established by our previous study of Taiwanese children (1). First, the Bayley-III cognitive and language raw scores were compiled into mental raw scores based on their corresponding weights in the BSID-II. A linear regression model was then established to convert the Bayley-III raw scores into the BSID-II raw scores for the individual scale at 6 and 12 months of age, respectively, in which preterm birth and sex were treated as covariates (*r^2^* = 0.62 to 0.63 for the mental scores at 6 and 12 months; *r^2^* = 0.58 to 0.74 for the motor scores at 6 and 12 months). Finally, the regression coefficients were applied to derive the estimated BSID-II raw scores for each child in the replication sample.

**References**

1. Wu YT, Tsou KI, Hsu CH, Fang LJ, Yao G, Jeng SF. Taiwanese infants' mental and motor development - 6-24 months. *J Pediatr Psychol.* (2008) 33:102-108

2. Yu YT, Hsieh WS, Hsu CH, Lin YJ, Lin CH, Hsieh S, Lu L, Cherng RJ, Chang YJ, Fan PC, Yao NJ, Chen WJ, Jeng SF. Family-centered care improved neonatal medical and neurobehavioral outcomes in preterm infants: randomized controlled trial. *Phys Ther.* (2017) 97:1158-1168

3. Bayley N. Bayley Scales of Infant Development. San Antonio, TX: *The Psychological Corporation, Harcourt Brace & Company*. (1993).

4. Bayley N, Reuner G. Bayley Scales of Infant and Toddler Development: Bayley-III. San Antonio, TX, USA: *Harcourt Assessment Inc.* (2006).

**SUPPLEMENTARY TABLES**

**Table S1.** Comparison of developmental scores of cohorts in preterm and term children, respectively

|  |  | Preterm (N = 201) | | | | | |  |  | Term (N = 111) | | | | | |
| --- | --- | --- | --- | --- | --- | --- | --- | --- | --- | --- | --- | --- | --- | --- | --- |
|  | Mental raw score | | |  | Motor raw score | | |  | Mental raw score | | |  | Motor raw score | | |
| Age | Cohort I | Cohort II | Cohort III |  | Cohort I | Cohort II | Cohort III |  | Cohort I | Cohort II | Cohort III |  | Cohort I | Cohort II | Cohort III |
| 6M | 57(4) | 59(4) | 57(4) |  | 34(4) | 36(4) | 34(4) |  | 58(3) | -- | 59(2) |  | 36(3) | -- | 36(3) |
| 12M | 79(4)^ab^ | 82(3)^a^ | 83(3)^b^ |  | 59(2) | 58(3) | 59(2) |  | 84(3)^a^ | -- | 85(3)^a^ |  | 62(2)^a^ | -- | 60(2)^a^ |
| 18M | 102(5)^a^ | 106(7)^a^ | 104(5) |  | 71(2) | 72(3) | 71(3) |  | 109(5) | -- | 107(5) |  | 73(2)^a^ | -- | 72(2)^a^ |
| 24M | 123(8)^a^ | 128(8) | 127(7)^a^ |  | 81(3) | 82(3) | 80(3) |  | 132(6) | -- | 130(7) |  | 84(3)^a^ | -- | 82(3)^a^ |
| 36M | 150(5) | 149(8) | 151(5) |  | 97(4) | 97(4) | 96(3) |  | 158(5) | 159(4)^a^ | 155(4)^a^ |  | 100(3)^a^ | 103(2)^ab^ | 99(3)^b^ |

*Note.* Data are presented as Mean(SD).

^ab^ *p* < .05 in comparing developmental scores between different cohort using analysis of variance (ANOVA).

**Table S2.** Distribution for the 15 SNPs of dopamine-related genes

| Gene | SNP (allele 1/2) | Preterm | |  | Term | |  | Preterm (Replication) | |
| --- | --- | --- | --- | --- | --- | --- | --- | --- | --- |
|  |  | Presence of allele 1  N (%) | Absence of allele 1  N (%) |  | Presence of allele 1  N (%) | Absence of allele 1  N (%) |  | Presence of allele 1  N (%) | Absence of allele 1  N (%) |
| DRD2 | rs1800497 (G/A) | 170 (85) | 31 (15) |  | 94 (85) | 17 (15) |  | -- | -- |
| DRD3 | rs167771 (G/A)^a^ | 73 (36) | 128 (64) |  | 36 (32) | 75 (68) |  | -- | -- |
| DAT1 | rs27072 (T/C)^a^ | 103 (51) | 98 (49) |  | 50 (45) | 61 (55) |  | -- | -- |
| DAT1 | rs2550948 (T/C)^a^ | 65 (32) | 136 (68) |  | 38 (34) | 73 (66) |  | -- | -- |
| COMT | rs4818 (C/G) | 150 (75) | 49 (25) |  | 86 (77) | 25 (23) |  | -- | -- |
| COMT | rs4680 (G/A) | 155 (77) | 46 (23) |  | 84 (76) | 27 (24) |  | -- | -- |
| COMT | rs2075507 (T/C) | 176 (88) | 25 (12) |  | 99 (89) | 12 (11) |  | -- | -- |
| MAOA | rs12843268 (T/C) | 134 (67) | 67 (33) |  | 72 (65) | 39 (35) |  | -- | -- |
| MAOA | rs2072744 (T/C) | 133 (68) | 64 (32) |  | 73 (66) | 38 (34) |  | -- | -- |
| MAOA | rs5905859 (C/A) | 134 (67) | 67 (33) |  | 73 (67) | 36 (33) |  | -- | -- |
| MAOA | rs3027400 (T/G) | 133 (66) | 68 (34) |  | 73 (66) | 38 (34) |  | -- | -- |
| MAOA | rs2235186 (A/G) | 133 (66) | 68 (34) |  | 73 (66) | 38 (34) |  | -- | -- |
| MAOA | rs2235185 (A/G) | 131 (66) | 67 (34) |  | 67 (65) | 36 (35) |  | -- | -- |
| MAOA | rs2239448 (T/C) | 135 (67) | 66 (33) |  | 73 (66) | 38 (34) |  | 197 (77) | 59 (23) |
| MAOA | rs3027407 (A/G) | 134 (67) | 66 (33) |  | 73 (66) | 38 (34) |  | -- | -- |

*Note.* Data presented as number (%). DRD2/3 = Dopamine D2/D3 receptors; DAT = Dopamine transporter; COMT = Catechol-O-methyltransferase; MAOA = monoamine oxidase A.

^a^ Determined by collapsing two adjacent genotypes due to one homozygous group having a very small number.

**Table S3.** Relations of genotype, preterm birth and age trend with the mental raw scores in the learning sample using three-way models (cohort, intervention, and sex treated as covariates)

|  |  | Age trend | |  | Genotype | |  | Preterm | |  | Genotype × Age trend | |  | Genotype × Preterm | |  | Preterm × Age trend | |  | Genotype × Preterm × Age trend | |
| --- | --- | --- | --- | --- | --- | --- | --- | --- | --- | --- | --- | --- | --- | --- | --- | --- | --- | --- | --- | --- | --- |
| Gene | SNP | F value | p |  | F value | p |  | F value | p |  | F value | p |  | F value | p |  | F value | p |  | F value | p |
| DRD2 | rs1800497 | 8935 | <0.0001^b^ |  | 2.08 | 0.15 |  | 36.32 | <0.0001^b^ |  | 0.75 | 0.56 |  | 0.00 | 0.99 |  | 4.15 | 0.0025 ^b^ |  | 1.66 | 0.16 |
| DRD3 | rs167771^a^ | 15733 | <0.0001^b^ |  | 1.22 | 0.27 |  | 55.45 | <0.0001^b^ |  | 1.09 | 0.36 |  | 0.40 | 0.53 |  | 8.50 | <0.0001^b^ |  | 0.84 | 0.50 |
| DAT1 | rs27072^a^ | 16804 | <0.0001^b^ |  | 1.34 | 0.25 |  | 60.55 | <0.0001^b^ |  | 0.18 | 0.95 |  | 0.88 | 0.35 |  | 10.28 | <0.0001^b^ |  | 0.33 | 0.86 |
| DAT1 | rs2550948^a^ | 15068 | <0.0001^b^ |  | 1.49 | 0.22 |  | 56.51 | <0.0001^b^ |  | 0.24 | 0.91 |  | 0.24 | 0.91 |  | 8.30 | <0.0001^b^ |  | 0.71 | 0.58 |
| COMT | rs4818 | 10248 | <0.0001^b^ |  | 0.55 | 0.46 |  | 51.68 | <0.0001^b^ |  | 1.08 | 0.37 |  | 0.47 | 0.49 |  | 9.97 | <0.0001^b^ |  | 1.56 | 0.19 |
| COMT | rs4680 | 11619 | <0.0001^b^ |  | 4.70 | 0.03 |  | 47.96 | <0.0001^b^ |  | 2.63 | 0.033 |  | 0.07 | 0.79 |  | 9.98 | <0.0001^b^ |  | 1.03 | 0.39 |
| COMT | rs2075507 | 6826 | <0.0001^b^ |  | 0.30 | 0.59 |  | 27.27 | <0.0001^b^ |  | 0.79 | 0.53 |  | 0.00 | 0.96 |  | 7.08 | <0.0001^b^ |  | 1.49 | 0.20 |
| MAOA | rs2239448 | 15245 | <0.0001^b^ |  | 1.45 | 0.23 |  | 70.94 | <0.0001^b^ |  | 4.85 | 0.0007^b^ |  | 6.60 | 0.010 |  | 10.39 | <0.0001^b^ |  | 1.27 | 0.28 |

*Note.* DRD2/3 = Dopamine D2/D3 receptors; DAT = Dopamine transporter; COMT = Catechol-O-methyltransferase; MAOA = monoamine oxidase A.

^a^ Determined by collapsing two adjacent genotypes due to one homozygous group having a very small number.

^b^ The significance levels reached the false discovery rate thresholds for 56 *p-*values.

**Table S4.** Relations of genotype, preterm birth and age trend with the mental raw scores in the learning sample using two-way models (cohort, intervention and sex treated as covariates)

|  |  | Age trend | |  | Genotype | |  | Preterm | |  | Genotype × Age trend | |  | Genotype × Preterm | |  | Preterm × Age trend | |
| --- | --- | --- | --- | --- | --- | --- | --- | --- | --- | --- | --- | --- | --- | --- | --- | --- | --- | --- |
| Gene | SNP (allele 1/2) | F value | p |  | F value | p |  | F value | p |  | F value | p |  | F value | p |  | F value | p |
| DRD2 | rs1800497 (G/A) | 9646.67 | <0.0001^b^ |  | 1.91 | 0.17 |  | 37.01 | <0.0001^b^ |  | 0.83 | 0.51 |  | 0.01 | 0.92 |  | 10.14 | <0.0001^b^ |
| DRD3 | rs167771 (G/A)^a^ | 15935.2 | <0.0001^b^ |  | 1.11 | 0.29 |  | 55.31 | <0.0001^b^ |  | 1.41 | 0.23 |  | 0.42 | 0.52 |  | 10.32 | <0.0001^b^ |
| DAT1 | rs27072 (T/C)^a^ | 16850.7 | <0.0001^b^ |  | 1.22 | 0.27 |  | 61.37 | <0.0001^b^ |  | 0.15 | 0.96 |  | 0.97 | 0.32 |  | 10.30 | <0.0001^b^ |
| DAT1 | rs2550948 (T/C)^a^ | 15269.5 | <0.0001^b^ |  | 1.25 | 0.26 |  | 55.96 | <0.0001^b^ |  | 0.30 | 0.88 |  | 0.02 | 0.89 |  | 10.22 | <0.0001^b^ |
| COMT | rs4818 (C/G) | 11643.5 | <0.0001^b^ |  | 0.50 | 0.48 |  | 52.25 | <0.0001^b^ |  | 0.33 | 0.86 |  | 0.35 | 0.56 |  | 10.03 | <0.0001^b^ |
| COMT | rs4680 (G/A) | 12177.6 | <0.0001^b^ |  | 4.96 | 0.03 |  | 48.39 | <0.0001^b^ |  | 2.09 | 0.08 |  | 0.07 | 0.79 |  | 10.49 | <0.0001^b^ |
| COMT | rs2075507 (T/C) | 7666.10 | <0.0001^b^ |  | 0.49 | 0.49 |  | 28.48 | <0.0001^b^ |  | 0.70 | 0.59 |  | 0.00 | 0.96 |  | 10.37 | <0.0001^b^ |
| MAOA | rs2239448 (T/C) | 15366.4 | <0.0001^b^ |  | 1.66 | 0.20 |  | 70.45 | <0.0001^b^ |  | 7.88 | <0.0001^b^ |  | 6.00 | 0.015 |  | 10.73 | <0.0001^b^ |

*Note.* DRD2/3 = Dopamine D2/D3 receptors; DAT = Dopamine transporter; COMT = Catechol-O-methyltransferase; MAOA = monoamine oxidase A.

^a^ Determined by collapsing two adjacent genotypes due to one homozygous group having a very small number.

^b^ The significance levels reached the false discovery rate thresholds for 90 *p-*values. There was no significant effect in the analysis of all the three-way interactions (Genotype × Preterm × Age trend) therefore we refit the model without the three-way interaction.

**Table S5.** Mental raw scores in preterm and term children in learning sample at 6, 12, 18, 24 and 36 months of age

|  | Preterm | |  | Term | |
| --- | --- | --- | --- | --- | --- |
| Variable (allele 1/2) | Presence of allele 1 | Absence of allele 1 |  | Presence of allele 1 | Absence of allele 1 |
| rs1800497 (G/A) | N=170 | N=31 |  | N=94 | N=17 |
| 6M | 57.5 (0.5) | 55.7 (0.7) |  | 59.7 (0.5) | 60.6 (0.8) |
| 12M | 82.3 (0.5) | 81.7 (0.8) |  | 85.8 (0.5) | 86.0 (0.9) |
| 18M | 104.2 (0.7) | 105.1 (1.1) |  | 109.3 (0.7) | 108.2 (1.4) |
| 24M | 126.6 (0.9) | 126.4 (1.4) |  | 132.2 (0.7) | 128.4 (1.6) |
| 36M | 150.3 (0.8) | 148.7 (1.2) |  | 157.5 (0.5) | 155.8 (1.3) |
| rs167771 (G/A) ^a^ | N=73 | N=128 |  | N=75 | N=36 |
| 6M | 57.5 (4.0) | 57.8 (4.6) |  | 59.1 (2.5) | 59.2 (2.9) |
| 12M | 82.8 (4.1) | 82.2 (4.2) |  | 86.2 (3.3) | 84.6 (3.5) |
| 18M | 105.8 (6.8) | 104.2 (5.9) |  | 108.9 (6.9) | 108.4 (4.6) |
| 24M | 128.1 (8.1) | 126.9 (8.1) |  | 131.1 (7.7) | 131.3 (6.7) |
| 36M | 151.3 (6.8) | 150.4 (6.3) |  | 157.0 (5.5) | 157.5 (5.2) |
| rs27072 (T/C) ^a^ | N=103 | N=98 |  | N=50 | N=61 |
| 6M | 58.0 (4.5) | 57.4 (4.3) |  | 58.9 (3.1) | 59.5 (2.4) |
| 12M | 82.9 (3.8) | 81.9 (4.5) |  | 85.1 (3.8) | 85.2 (3.2) |
| 18M | 105.1 (6.6) | 104.5 (6.0) |  | 108.9 (5.7) | 108.2 (5.5) |
| 24M | 128.0 (8.2) | 126.7 (7.9) |  | 131.1 (7.5) | 131.4 (6.7) |
| 36M | 151.4 (6.1) | 150.0 (6.8) |  | 157.1 (5.8) | 157.5 (4.9) |
| rs2550948 (T/C) ^a^ | N=65 | N=136 |  | N=38 | N=73 |
| 6M | 57.8 (4.2) | 57.7 (4.5) |  | 59.5 (2.8) | 59.0 (2.8) |
| 12M | 82.9 (4.6) | 82.3 (3.9) |  | 85.8 (3.5) | 84.8 (3.5) |
| 18M | 104.7 (6.5) | 104.9 (6.2) |  | 109.9 (5.1) | 107.9 (5.7) |
| 24M | 128.1 (7.9) | 127.0 (8.2) |  | 131.7 (7.6) | 131.0 (6.8) |
| 36M | 151.3 (5.9) | 150.4 (6.8) |  | 157.5 (5.6) | 157.3 (5.1) |

**Table S5.** (cont. [I])

|  | Preterm | |  | Term | |
| --- | --- | --- | --- | --- | --- |
| Variable (allele 1/2) | Presence of allele 1 | Absence of allele 1 |  | Presence of allele 1 | Absence of allele 1 |
| rs4818 (C/G) | N=150 | N=49 |  | N=86 | N=25 |
| 6M | 57.1 (0.5) | 57.4 (0.7) |  | 59.9 (0.5) | 59.7 (0.9) |
| 12M | 82.1 (0.6) | 82.1 (0.7) |  | 86.0 (0.5) | 85.1 (0.9) |
| 18M | 104.3 (0.7) | 104.2 (0.9) |  | 109.0 (0.7) | 109.5 (1.4) |
| 24M | 126.7 (0.9) | 125.4 (1.2) |  | 130.9 (0.7) | 134.7 (1.6) |
| 36M | 150.8 (0.9) | 149.3 (1.1) |  | 157.2 (0.6) | 157.5 (1.0) |
| rs4680 (G/A) | N=155 | N=46 |  | N=84 | N=27 |
| 6M | 56.9 (0.6) | 57.9 (0.7) |  | 59.9 (0.5) | 59.0 (0.8) |
| 12M | 82.1 (0.6) | 82.0 (0.7) |  | 85.8 (0.5) | 85.9 (0.8) |
| 18M | 103.8 (0.7) | 105.5 (0.9) |  | 108.4 (0.7) | 111.6 (1.3) |
| 24M | 126.2 (0.9) | 127.9 (1.2) |  | 130.9 (0.8) | 133.5 (1.5) |
| 36M | 149.7 (0.8) | 150.6 (1.1) |  | 156.8 (0.6) | 158.7 (1.0) |
| rs2075507 (T/C) | N=176 | N=25 |  | N=99 | N=12 |
| 6M | 57.1 (0.5) | 57.6 (0.9) |  | 60.0 (0.5) | 58.3 (1.0) |
| 12M | 82.2 (0.5) | 81.7 (1.0) |  | 85.6 (0.5) | 88.1 (1.1) |
| 18M | 104.2 (0.7) | 105.1 (1.2) |  | 109.0 (0.6) | 110.3 (1.8) |
| 24M | 126.3 (0.8) | 127.8 (1.5) |  | 131.4 (0.7) | 132.4 (2.1) |
| 36M | 150.1 (0.8) | 148.6 (1.5) |  | 157.1 (0.5) | 158.4 (1.4) |
| rs12843268 (T/C) | N=134 | N=67 |  | N=72 | N=39 |
| 6M | 57.2 (0.6) | 56.8 (0.6) |  | 59.6 (0.5) | 60.4 (0.6) |
| 12M | 82.5 (0.6) | 81.3 (0.6) |  | 85.5 (0.5) | 86.7 (0.7) |
| 18M | 105.2 (0.7) | 102.1 (0.8) |  | 109.0 (0.7) | 109.4 (1.0) |
| 24M | 128.0 (1.0) | 123.4 (1.0) |  | 131.9 (0.8) | 131.0 (1.1) |
| 36M | 150.6 (0.9) | 148.6 (0.9) |  | 157.4 (0.6) | 157.0 (0.8) |

**Table S5.** (cont. [II])

|  | Preterm | |  | Term | |
| --- | --- | --- | --- | --- | --- |
| Variable (allele 1/2) | Presence of allele 1 | Absence of allele 1 |  | Presence of allele 1 | Absence of allele 1 |
| rs2072744 (T/C) | N=133 | N=64 |  | N=73 | N=38 |
| 6M | 57.3 (0.6) | 57.0 (0.6) |  | 59.6 (0.5) | 60.5 (0.6) |
| 12M | 82.5 (0.6) | 81.3 (0.6) |  | 85.5 (0.5) | 86.7 (0.7) |
| 18M | 105.3 (0.7) | 101.9 (0.8) |  | 109.1 (0.7) | 109.3 (1.0) |
| 24M | 128.1 (1.0) | 123.0 (1.0) |  | 131.9 (0.8) | 130.8 (1.1) |
| 36M | 150.6 (0.9) | 148.4 (0.9) |  | 157.5 (0.6) | 156.8 (0.8) |
| rs5905859 (C/A) | N=134 | N=67 |  | N=73 | N=36 |
| 6M | 57.3 (0.6) | 56.9 (0.6) |  | 59.7 (0.5) | 60.3 (0.6) |
| 12M | 82.5 (0.6) | 81.4 (0.6) |  | 85.7 (0.5) | 86.5 (0.7) |
| 18M | 105.2 (0.7) | 102.2 (0.8) |  | 109.4 (0.7) | 109.0 (1.0) |
| 24M | 128.2 (1.0) | 123.3 (1.0) |  | 132.1 (0.8) | 130.6 (1.2) |
| 36M | 150.7 (0.9) | 148.5 (0.9) |  | 157.4 (0.6) | 156.8 (0.8) |
| rs3027400 (T/G) | N=133 | N=68 |  | N=73 | N=38 |
| 6M | 57.3 (0.6) | 56.9 (0.6) |  | 59.6 (0.5) | 60.5 (0.6) |
| 12M | 82.5 (0.6) | 81.4 (0.6) |  | 85.5 (0.5) | 86.7 (0.7) |
| 18M | 105.2 (0.7) | 102.2 (0.8) |  | 109.1 (0.7) | 109.3 (1.0) |
| 24M | 128.2 (1.0) | 123.3 (1.0) |  | 131.9 (0.8) | 130.8 (1.1) |
| 36M | 150.7 (0.9) | 148.5 (0.9) |  | 157.5 (0.6) | 156.8 (0.8) |
| rs2235186 (A/G) | N=133 | N=68 |  | N=73 | N=38 |
| 6M | 57.3 (0.6) | 56.8 (0.5) |  | 59.6 (0.5) | 60.5 (0.6) |
| 12M | 82.5 (0.6) | 81.4 (0.6) |  | 85.5 (0.5) | 86.7 (0.7) |
| 18M | 105.2 (0.7) | 102.2 (0.7) |  | 109.1 (0.7) | 109.3 (1.0) |
| 24M | 128.2 (1.0) | 123.3 (0.9) |  | 131.9 (0.8) | 130.8 (1.1) |
| 36M | 150.7 (0.9) | 148.5 (0.8) |  | 157.5 (0.6) | 156.8 (0.8) |

**Table S5.** (cont. [III])

|  | Preterm | |  | Term | |
| --- | --- | --- | --- | --- | --- |
| Variable (allele 1/2) | Presence of allele 1 | Absence of allele 1 |  | Presence of allele 1 | Absence of allele 1 |
| rs2235185 (A/G) | N=131 | N=67 |  | N=67 | N=36 |
| 6M | 57.5 (0.6) | 56.9 (0.6) |  | 59.9 (0.5) | 60.7 (0.6) |
| 12M | 82.6 (0.6) | 81.5 (0.6) |  | 85.8 (0.5) | 86.9 (0.7) |
| 18M | 105.5 (0.8) | 102.3 (0.7) |  | 109.4 (0.8) | 109.5 (1.0) |
| 24M | 127.9 (1.0) | 123.6 (1.0) |  | 132.4 (0.9) | 130.9 (1.1) |
| 36M | 150.6 (0.9) | 149.3 (0.9) |  | 158.0 (0.6) | 156.8 (0.8) |
| rs2239448 (T/C) | N=135 | N=66 |  | N=73 | N=38 |
| 6M | 57.3 (0.5) | 56.8 (0.6) |  | 59.6 (0.5) | 60.5 (0.6) |
| 12M | 82.5 (0.6) | 81.4 (0.6) |  | 85.5 (0.5) | 86.7 (0.7) |
| 18M | 105.3 (0.7) | 102.1 (0.7) |  | 109.1 (0.7) | 109.3 (1.0) |
| 24M | 128.2 (1.0) | 123.0 (1.0) |  | 131.9 (0.8) | 130.8 (1.1) |
| 36M | 150.8 (0.9) | 148.3 (0.9) |  | 157.5 (0.6) | 156.8 (0.8) |
| rs3027407 (A/G) | N=134 | N=66 |  | N=73 | N=38 |
| 6M | 57.3 (0.5) | 56.7 (0.6) |  | 59.6 (0.5) | 60.5 (0.6) |
| 12M | 82.5 (0.6) | 81.3 (0.6) |  | 85.5 (0.5) | 86.7 (0.7) |
| 18M | 105.2 (0.7) | 102.1 (0.8) |  | 109.1 (0.7) | 109.3 (1.0) |
| 24M | 128.2 (1.0) | 123.1 (1.0) |  | 131.9 (0.8) | 130.8 (1.1) |
| 36M | 150.8 (0.7) | 148.3 (0.9) |  | 157.5 (0.6) | 156.8 (0.8) |

*Note.* Data presents as Mean (SD).

^a^ Determined by collapsing two adjacent genotypes due to one homozygous group having a very small number.

**Table S6.** Relations of genotypes with the mental raw scores at 6, 12, 18, 24 and 36 months of age in term children of the learning sample (cohort, intervention and sex are treated as covariates)

| Gene | SNP (allele 1/2) | Difference in metal score (presence of allele 1 - absence of allele 1) | | | | |  | Age trend | |  | Genotype | |  | Genotype × Age trend | |
| --- | --- | --- | --- | --- | --- | --- | --- | --- | --- | --- | --- | --- | --- | --- | --- |
|  |  | 6 months | 12 months | 18 months | 24 months | 36 months |  | F value | p |  | F value | p |  | F value | p |
| DRD2 | rs1800497 (G/A) | -0.90 | -0.20 | 1.10 | 3.80 | 1.70 |  | 3621 | <0.0001^b^ |  | 1.53 | 0.22 |  | 1.22 | 0.31 |
| DRD3 | rs167771 (G/A)^a^ | -1.50 | -0.80 | -3.20 | -0.50 | 1.40 |  | 1171 | <0.0001^b^ |  | 0.47 | 0.49 |  | 0.62 | 0.65 |
| DAT1 | rs27072 (T/C)^a^ | -1.30 | -0.20 | 0.70 | 0.10 | 3.90 |  | 737 | <0.0001^b^ |  | 0.01 | 0.91 |  | 0.42 | 0.79 |
| DAT1 | rs2550948 (T/C)^a^ | -2.30 | 1.50 | 2.60 | -2.40 | -3.70 |  | 783 | <0.0001^b^ |  | 0.02 | 0.90 |  | 0.94 | 0.44 |
| COMT | rs4818 (C/G) | 0.20 | 0.90 | -0.50 | -3.80 | -0.30 |  | 3872 | <0.0001^b^ |  | 0.57 | 0.45 |  | 2.69 | 0.031 |
| COMT | rs4680 (G/A) | 0.90 | -0.10 | -3.20 | -2.60 | -1.90 |  | 4731 | <0.0001^b^ |  | 1.86 | 0.17 |  | 2.37 | 0.052 |
| COMT | rs2075507 (T/C) | 1.70 | -2.50 | -1.30 | -1.00 | -1.30 |  | 2749 | <0.0001^b^ |  | 0.20 | 0.65 |  | 1.16 | 0.33 |
| MAOA | rs2239448 (T/C) | -0.90 | -1.20 | -0.20 | 1.10 | 0.70 |  | 6005 | <0.0001^b^ |  | 1.06 | 0.31 |  | 0.82 | 0.51 |

*Note.* Data presented as model-based estimated mean (SD). DRD2/3 = Dopamine D2/D3 receptors; DAT = Dopamine transporter; COMT = Catechol-O-methyltransferase; MAOA = monoamine oxidase A.

^a^ Determined by collapsing two adjacent genotypes due to one homozygous group having a very small number.

^b^ The significance levels reached the Bonferroni correction thresholds for 24 *p-*values.

**Table S7.** Relations of genotype, preterm birth and age trend with the motor raw scores in the learning sample using three-way models (cohort, intervention and sex treated as covariates)

|  |  | Age trend | |  | Genotype | |  | Preterm | |  | Genotype × Age trend | |  | Genotype × Preterm | |  | Preterm × Age trend | |  | Genotype × Preterm  × Age trend | |
| --- | --- | --- | --- | --- | --- | --- | --- | --- | --- | --- | --- | --- | --- | --- | --- | --- | --- | --- | --- | --- | --- |
| Gene | SNP (allele 1/2) | F value | p |  | F value | p |  | F value | p |  | F value | p |  | F value | p |  | F value | p |  | F value | p |
| DRD2 | rs1800497 (G/A) | 9643.57 | <0.0001^b^ |  | 0.65 | 0.42 |  | 34.81 | <0.0001^b^ |  | 1.33 | 0.26 |  | 0.00 | 0.98 |  | 1.78 | 0.13 |  | 1.32 | 0.26 |
| DRD3 | rs167771 (G/A)^a^ | 16525.2 | <0.0001^b^ |  | 0.08 | 0.78 |  | 51.79 | <0.0001^b^ |  | 0.76 | 0.55 |  | 0.38 | 0.54 |  | 5.16 | 0.0004^b^ |  | 0.64 | 0.63 |
| DAT1 | rs27072 (T/C)^a^ | 17786.5 | <0.0001^b^ |  | 1.54 | 0.22 |  | 58.00 | <0.0001^b^ |  | 0.50 | 0.74 |  | 0.01 | 0.93 |  | 5.14 | 0.0004^b^ |  | 1.28 | 0.27 |
| DAT1 | rs2550948 (T/C)^a^ | 15876.8 | <0.0001^b^ |  | 0.05 | 0.82 |  | 54.12 | <0.0001^b^ |  | 0.48 | 0.75 |  | 0.01 | 0.91 |  | 4.29 | 0.0019 |  | 0.19 | 0.94 |
| COMT | rs4818 (C/G) | 10837.0 | <0.0001^b^ |  | 0.00 | 0.99 |  | 41.30 | <0.0001^b^ |  | 1.40 | 0.23 |  | 0.08 | 0.77 |  | 5.66 | 0.0002^b^ |  | 1.74 | 0.14 |
| COMT | rs4680 (G/A) | 12044.0 | <0.0001^b^ |  | 5.47 | 0.02 |  | 49.29 | <0.0001^b^ |  | 0.70 | 0.59 |  | 0.54 | 0.46 |  | 5.26 | 0.0003^b^ |  | 0.62 | 0.65 |
| COMT | rs2075507 (T/C) | 7075.96 | <0.0001^b^ |  | 0.75 | 0.39 |  | 35.55 | <0.0001^b^ |  | 0.46 | 0.77 |  | 1.20 | 0.27 |  | 3.47 | 0.0080 |  | 0.36 | 0.84 |
| MAOA | rs2239448 (T/C) | 15709.7 | <0.0001^b^ |  | 0.26 | 0.61 |  | 60.90 | <0.0001^b^ |  | 1.90 | 0.11 |  | 2.00 | 0.16 |  | 5.21 | 0.0004^b^ |  | 0.48 | 0.75 |

*Note.* DRD2/3 = Dopamine D2/D3 receptors; DAT = Dopamine transporter; COMT = Catechol-O-methyltransferase; MAOA = monoamine oxidase A.

^a^ Determined by collapsing two adjacent genotypes due to one homozygous group having a very small number.

^b^ The significance levels reached the Bonferroni correction thresholds for 56 p-values.

**Table S8.** Exploring the combinations of rs2239448 with other SNPs on the analysis of genotype, preterm birth, and age trend with the mental raw scores in the learning sample using three-way models (cohort, intervention and sex treated as covariates)

|  | Age trend | |  | Genotype | |  | Preterm | |  | Genotype × Age trend | |  | Genotype × Preterm | |  | Preterm × Age trend | |  | Genotype × Preterm × Age trend | |
| --- | --- | --- | --- | --- | --- | --- | --- | --- | --- | --- | --- | --- | --- | --- | --- | --- | --- | --- | --- | --- |
| Number of SNPs | F value | p |  | F value | p |  | F value | p |  | F value | p |  | F value | p |  | F value | p |  | F value | p |
| 1 (rs2239448) | 15245.5 | <0.0001^a^ |  | 1.45 | 0.23 |  | 70.94 | <0.0001^a^ |  | 4.85 | 0.0007^a^ |  | 6.60 | 0.010 |  | 10.39 | <0.0001^a^ |  | 1.27 | 0.28 |
| 2 SNPs | 8315.59 | <0.0001^a^ |  | 0.55 | 0.58 |  | 45.54 | <0.0001^a^ |  | 0.87 | 0.54 |  | 2.16 | 0.12 |  | 7.56 | <0.0001^a^ |  | 1.02 | 0.42 |
| 3 SNPs | 8441.25 | <0.0001^a^ |  | 0.14 | 0.94 |  | 46.81 | <0.0001^a^ |  | 0.56 | 0.87 |  | 1.30 | 0.27 |  | 7.83 | <0.0001^a^ |  | 1.05 | 0.40 |
| 4 SNPs | 4147.01 | <0.0001^a^ |  | 1.72 | 0.14 |  | 22.55 | <0.0001^a^ |  | 0.94 | 0.52 |  | 1.05 | 0.38 |  | 7.69 | <0.0001^a^ |  | 1.71 | 0.04 |
| 5 SNPs | 7253.56 | <0.0001^a^ |  | 1.70 | 0.15 |  | 44.30 | <0.0001^a^ |  | 0.54 | 0.93 |  | 0.95 | 0.43 |  | 9.37 | <0.0001^a^ |  | 1.59 | 0.07 |
| 6 SNPs | 9991.67 | <0.0001^a^ |  | 1.13 | 0.34 |  | 48.67 | <0.0001^a^ |  | 1.11 | 0.34 |  | 0.77 | 0.54 |  | 11.97 | <0.0001^a^ |  | 1.07 | 0.38 |
| 7 SNPs | 8193.16 | <0.0001^a^ |  | 1.58 | 0.16 |  | 41.68 | <0.0001^a^ |  | 1.04 | 0.41 |  | 0.75 | 0.59 |  | 6.57 | <0.0001^a^ |  | 1.07 | 0.38 |
| 8 SNPs | 6519.29 | <0.0001^a^ |  | 1.51 | 0.17 |  | 43.14 | <0.0001^a^ |  | 1.60 | 0.03 |  | 1.48 | 0.18 |  | 6.72 | <0.0001^a^ |  | 1.63 | 0.03 |

*Note.* The combination of MAOA marker rs2239448 and other seven markers were according to the ascending order of *p*-value (i.e., rs2239448, rs4680, rs167771, rs4818, rs2075507, rs1800497, rs27072, and rs2550948). SNP = Single nucleotide polymorphism.

^a^ The significance levels reached the Bonferroni correction thresholds for 56 *p-*values.

**SUPPLEMENTARY FIGURES**

Recruitment

Cohort I: Preterm children (N = 158) and term children (N = 113)

Cohort II: Preterm children (N = 78) and term children (N = 34)

Cohort III: Preterm children (N = 178) and term children (N = 63)

Cohort IV: Preterm children (N = 275)

Developmental data
(Developmental measurement from 6 to 36 months of age)

Cohort I: Preterm children (N = 112) and term children (N = 102)

Cohort II: Preterm children (N = 73) and term children (N = 30)

Cohort III: Preterm children (N = 173) and term children (N = 59)

(Developmental measurement from 6 to 36 months of age)

Cohort IV: Preterm children (N=275)

Genetic data
(Collected buccal cell sample at school age)

Cohort I: Preterm children (N = 40) and term children (N = 39)

Cohort II: Preterm children (N = 41) and term children (N = 29)

Cohort III: Preterm children (N = 138) and term children (N = 50)

Cohort IV: Preterm children (N=256)

Children with both developmental and genetic data

Cohort I: Preterm children (N = 27) and term children (N = 38)

Cohort II: Preterm children (N = 39) and term children (N = 24)

Cohort III: Preterm children (N = 135) and term children (N = 49)

Cohort IV: Preterm children (N=256)

**Figure S1.** Flow chart of allocation and follow-up in preterm children and term children among each cohort


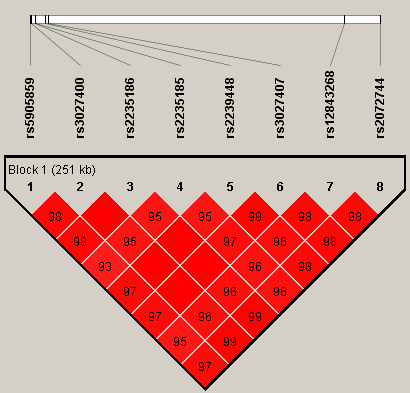


**Figure S2.** Haplotype and linkage disequilibrium plot of markers in MAOA gene in Cohort I-III (N = 312)
